# Internet addiction in students from an educational institution in Southern Brazil: prevalence and associated factors

**DOI:** 10.1590/2237-6089-2019-0098

**Published:** 2020-05-27

**Authors:** Gisele Bartz de Ávila, Érico Nobre dos Santos, Karen Jansen, Fernando Celso Barros

**Affiliations:** 1 Programa de Pós-Graduação em Saúde e Comportamento Universidade Católica de Pelotas PelotasRS Brazil Programa de Pós-Graduação em Saúde e Comportamento, Universidade Católica de Pelotas, Pelotas, RS, Brazil.; 2 Instituto Federal de Educação Tecnológica do Rio Grande do Sul PelotasRS Brazil Instituto Federal de Educação Tecnológica do Rio Grande do Sul, Pelotas, RS, Brazil.

**Keywords:** Internet, addiction, dependence, students, depression, anxiety

## Abstract

**Objective:**

To evaluate the prevalence of Internet addiction (IA) and its associated factors among students at an Educational Institution in Southern Brazil.

**Method:**

This is a cross-sectional study, targeting a sample of students aged from 14 to 20 years. They were selected by random sampling to be representative of the 4038 students enrolled at the institute at the time. IA was assessed using the Internet Addiction Test (IAT). Screening for anxiety and/or depressive disorders was performed using the Well-Being Index (WHO-5).

**Results:**

The prevalence of IA was 50.8% and the rate was higher among individuals who had screened positive for depressive or anxiety disorders than among those who had not (p = 0.003). There was an association between IA and access to certain types of content, such as gaming (p = 0.010), work and study related content (p = 0.030), and using the internet to access sexual content (p < 0.001).

**Conclusion:**

Further studies are needed to confirm the high prevalence of IA and explore factors associated with it in samples with similar characteristics to ours. The associations between this dependency and positive screening for anxiety and/or depressive disorders and the types of content accessed are an alert to the existence of these important relationships and illustrate the importance of studying them further. Knowledge about these associations provides an opportunity to implement measures for prevention, such as psychoeducation, and to offer adequate treatment.

## Introduction

The internet has become an important tool for social interaction and communication, as well as for accessing information, by creating a new virtual public space. As new forms of technology have been developed, it has also become more widespread and nowadays can be accessed via devices such as tablets and mobile phones. This communication network has unique features such as the capacity for quick and easy gratification, which, in addition to the increasing popularity of the Internet, makes it an instrument with the potential to generate psychological dependence. There is growing evidence of cases of individuals who suffer from internet addictive behaviors.^1 , 2^

One review has shown that the mesocorticolimbic reward system of subjects that experience internet addictive behaviors is impacted in the same manner as with substance abuse, and that the cue-induced craving phenomenon is likewise affected.^3^ The symptoms of excessive use of the internet have been found to be commonly associated with behavioral addictions and present neurological similarities to other addictions.^4^ Although the mechanisms of abuse of the internet and substance abuse can be similar, their consequences differ. According to Young,^5^ the physical risk factors involved with addiction to the internet are comparatively minimal, but detectable. To accommodate excessive use, sleep patterns are typically disrupted due to late night log-ins. This sleep deprivation causes excessive fatigue, often impairing academic or occupational functioning, and may affect the immune system, leaving the patient vulnerable to disease. Additionally, the sedentary element of prolonged computer use may result in a lack of proper exercise and lead to an increased risk of carpal tunnel syndrome, back strain, or eyestrain. While the physical side-effects of utilizing the internet are mild compared to chemical dependency, addictive use of the internet will result in similar familial, academic, and occupational impairments. The negative effects have an impact on the individual both at the intrapersonal and interpersonal level and can also occur in the form of tolerance/abstinence, denial, concealment/minimization of the problem, feelings of guilt, low self-esteem, and risk of relapse.^6 , 7^

Internet use has been identified as a problem for many years, since access became widespread, allowing its excessive or problematic use. The first proposal for Internet Addiction (IA) diagnostic criteria was presented by Young.^8^ Presence of five or more of the criteria was used as a cutoff to identify addicted internet users. These criteria include presence of an excessive concern with the internet; a usage pattern of between 40 and 80 hours per week; a need to increase online connected time to achieve the satisfaction desired; attempts to reduce time using the internet; presence of irritability and/or depression; presence of emotional lability when internet use is restricted; being online longer than scheduled; work and social relationships being at risk due to excessive use of the internet and; lying about the amount of time spent online. On the basis of these criteria, Young defines IA as an impulse control disturbance, which does not involve any kind of stimulant and is characterized by the existence of certain factors that influence the user to become dependent: gratifying emotions/sensations that are experienced through use (e.g. euphoria, excitement, relaxation, etc.); non-adaptive and catastrophic thoughts (e.g. low self-esteem, depression, etc.) which are attenuated through use; and life events that lead to addiction (e.g. disappointment with life, absence of intimate relationships, etc.). In general, addictive behaviors affect a large number of individuals who are unable to control the frequency with which they perform a certain behavior.^9 , 10^

In the literature, the most common terms used to describe the phenomenon are Internet addiction; problematic Internet use; pathological Internet use; and Internet abuse.^5 , 11 , 12^ This study adopts the term “Internet addiction” to refer to the subject because this was the first term to be used in the literature and is also the most often used for practical purposes.^5 , 13^ Studies around the world have found that young age is one of the risk factors most associated with IA,^12 , 14 - 16^ while one study identified that being a student is also a risk factor.^15^ The adolescent population is an IA risk group and their emotional-behavioral functioning includes elements of psychopathological risk that are specific to this phase. During adolescence, they start to psychologically separate from internalized parental figures and seek new objects outside the family and so their main developmental task therefore consists of construction of new identities.^17^ They appear to be particularly attracted by virtual networks in which it is possible to create a new coexistence space with proper communication characteristics, where anonymity and autonomy are intrinsic properties, and where they can develop their online identity indistinguishable from the offline one. This online identity and reputation are also felt to be crucial for handling of offline relationships.^17 , 18^ Griffiths states that internet use is highly associated with a perception that it is a way of dealing with and compensating for certain shortcomings, such as low self-esteem, making users feel better because the internet allows them to take on a different personality and social identity.^6^ Some adolescents can withdraw from real-life interactions by engaging in relationships through the internet. Through these relationships, which have become common with the development of social media, they can get support and recognition from peers and develop their social identity.^17^

The internet has certain particular characteristics that make it particularly attractive to the adolescent populations, such as: being infinite, offering services, leisure and social interactions at any time; providing continuous and immediate gratification subsequent to any action; being immersive, with the whole virtual environment constructed to attract, simulate and enhance the looks of the real world and; as stated above, providing community interdependence, low cost and anonymity, which encourage uninhibited actions.^17^

Previous studies have demonstrated a high level of comorbidity of IA with psychiatric symptoms in adolescent populations. One hypothesis is that IA could emerge as a result of individuals’ attempts to ‘self-treat’ their stress, anxiety, and depression symptoms.^19^ In addition to psychiatric problems, there are also other risk factors that have been associated with IA in adolescents, such as gender^20 - 22^ and the type of content accessed.^15 , 23^

A meta-analysis published in 2014 presented Internet Addiction indexes for 31 nations from seven world regions, showing an estimated global prevalence of 6.0%. The highest rates were seen in the Middle East (10.9%), North America (8%), Oceania (4.3%), Western Europe (2.6), Eastern Europe (6.1%), and Asia (7.1%). Only one country was analyzed in South America (0%), revealing a paucity of data for the region.^24^ Results are generally very heterogeneous, especially data on prevalence. This is partly due to the fact that the instruments used and the diagnostic criteria are not standardized and also due to sociocultural differences between the populations studied. Kuss et al.^13^ reviewed the epidemiological literature on IA and showed that studies using the Internet Addiction Test (IAT) reported prevalence rates ranging from 0.8% in Europe to 20.3% in South Korea. Since then, two studies using the same test have been conducted in Brazilian schools, reporting high IA scores. One evaluated the prevalence of IA among 91 adolescents aged from 12 to 16 in two schools in a Brazilian city, using a cross-sectional design. It reported a 21% prevalence of IA. Within the IA group, there was a positive correlation with anxiety and depression, among other problems.^18^ The other study investigated internet usage patterns and their relationships with anxiety and depression symptoms in a sample of 150 adolescents. They found a 61.3% risk of developing IA and did not find a correlation between the psychiatric symptoms and IAT scores.^25^ Both studies had non-probabilistic samples, selected by convenience, and the samples were not even representative of the schools from which they were recruited.

Data showing IA prevalence among adolescents from Brazil are scarce, and studies that do exist were conducted with few subjects, highlighting the need to uncover new evidence about IA and its risk factors within this population, from a larger, representative sample. Therefore, the present study aims to identify and evaluate IA presence and its associated factors among students at an educational institution in southern Brazil.

## Method

A cross-sectional study was conducted by administration of a self-report instrument to 14-to-20-year-old high school (technical courses), further education, and higher education students at Instituto Federal de Educação Tecnológica do Rio Grande do Sul, a Federal Institute of Technology in Southern Brazil. The sample size was estimated as 450 students, to be representative of the 4083 students enrolled at the institute at the time. Data were collected during the months of March and April 2017. Initially, the sample was randomly selected, picking one out of every five individuals on the student roll, and actively seeking them in their classrooms. Using this method, 137 questionnaires were answered. We then chose to select the remainder of the sample by random active search at the institution’s campus. This method gave all students an equal likelihood of selection. The rest of the sample was therefore selected using this procedure, totaling 342 students interviewed. Students were invited to participate in the study and the pertinent explanations were provided by a team of four psychologists and a psychiatrist. The study was approved by the ethics committee at the Universidade Católica de Pelotas and participants signed informed consent forms.

The questionnaire was self-administered and comprised 28 questions covering internet use patterns, socioeconomic classification, and items to screen for depressive and/or anxiety disorders. The socioeconomic classification was assessed using an economic classification scale published by Associação Brasileira de Empresas de Pesquisa (the Brazilian Association of research companies [ABEP]).^26^

### Internet Addiction Test (IAT)

The IAT-Portuguese version^1^ was used to assess Internet Addiction on the basis of DSM-IV diagnostic criteria for pathological gambling.^8^ The IAT is the instrument for IA assessment most widely-used in the world. It is a valid and reliable instrument for measuring the extent of a person’s involvement with the internet that classifies addictive behavior in terms of mild, moderate, or severe impairment. The IAT comprises 20 items, each of which is rated on a five-point Likert scale: “rarely or never” (1), “occasionally” (2), “frequently” (3), “often” (4), or “always” (5). The total IAT score is obtained by adding the scores for each response provided by the participant. The higher the score, the higher the level of addiction to the internet; normal range: 0-30 points, mild: 31- 49 points, moderate: 50-79 points, and severe: 80-100 points. The instrument’s author has suggested two different cut-off points criteria based on her opinion, considering scores in the range of 0-30 as indicating an absence of addiction.^8 , 24 , 27 , 28^ Because internet use via mobile devices has now become so widespread, questions have been added to the questionnaire to assess internet use by modality of usage (smartphones, tablets, computer) and the approximate time spent using each modality.

### Well-Being Index (WHO-5)

Presence of possible depressive and/or anxiety disorders was assessed using the WHO-5 questionnaire, which was created by the World Health Organization to measure quality of life in chronic patients. The five-item questionnaire was created in 1995 and has demonstrated efficacy for screening for depressive and anxiety disorders. The questionnaire has been validated in Portuguese^29^ and has accuracy of 0.87, sensitivity of 0.77, specificity of 0.89, positive predictive value of 0.81, and negative predictive value of 0.87, using a cutoff of 11 points. The questionnaire consists of five questions with a Likert response scale ranging from 0 to 3 and so the total score ranges from 0 to 15. The higher the score, the higher the measure of individual well-being. In this study, scores below 11 were considered positive for presence of depressive and/or anxious symptoms and disorders.

After coding the instruments, data were entered into the Stata/SE 13.0 program. Univariate analysis was performed to obtain simple frequencies and measures of central tendency and dispersion of variables. Pearson’s chi-square test and the Kruskal-Wallis test were used for bivariate analyses. Independent effects of variables on the outcome were estimated by multivariate analysis. Poisson regression was conducted with a hierarchical model. Variables with bivariate associations with the outcome with p < 0.20 were included in the multivariate model. A 5% (p < 0.05) significance level was adopted for all tests.

## Results

Table 1 describes the population studied. The sample comprised 327 individuals aged 14 to 20 years, 53.5% of whom were male, while 44.3% were aged 14 to 17 and 55.7% aged 18 to 20. The economic classification scale classified 59.6% belonged to the middle class - “C”. We assessed 80.9% of the subjects as screening positive for depression and/or anxiety disorders. With regard to the types of content accessed, 96.5% of the students reported that they use social networks often, very often, or always; 51.7% reported accessing the internet for games; 76% accessed it for news or information; 85.5% accessed the internet to work or study, and 20.2% accessed it for sexual content. In terms of internet use patterns, 49.2% of the population reported normal use, 34.9% exhibited a mild dependence pattern, and 15.9% displayed moderate to severe dependence. Only one student was rated as severely dependent. There was a gender difference between individuals who tested positive for depression and anxiety, with a higher frequency among females (86.8%) than males (75.7%; p = 0.016). Internet use for gaming was more frequent among males (65.5%) than females (35.6%; p < 0.001). Internet use for the purposes of information and news was higher among females (81.5%) than males (71.3%; p = 0.048).

**Table 1:** Description of the student sample

Variables	Distribution	Male	Female	p-value*
Age				0.517
14 to 17	145 (44.3)	81 (46.3)	64 (42.1)	
18 to 20	182 (55.7)	94 (53.7)	88 (57.9)	
Socioeconomic class				
High (A+B)	86 (26.5)	50 (28.9)	36 (23.8)	0.707
Middle (C)	193 (59.6)	97 (56.1)	96 (63.6)	
Low (D+E)	45 (13.9)	26 (15.0)	19 (12.6)	
Screened positive for depression and/or anxiety				0.016
No	62 (19.1)	42 (24.3)	20 (13.2)	
Yes	263 (80.9)	131 (75.7)	132 (86.8)	
Content accessed				
Social media	306 (96.5)	163 (95.3)	143 (97.9)	0.335
Games	164 (51.7)	112 (65.5)	52 (35.6)	< 0.001
Work or study	241 (76.0)	122 (71.3)	119 (81.5)	0.048
Information/news	271 (85.5)	144 (84.2)	127 (87.0)	0.590
Sexual content	64 (20.2)	53 (31.0)	11 (7.5)	< 0.001
Internet addiction				0.160
Normal use	161 (49.2)	82 (47.7)	76 (51.4)	
Mild dependence	114 (34.9)	56 (32.6)	57 (38.5)	
Moderate/severe dependence	52 (15.9)	34 (19.8)	15 (10.1)	
Total	327 (100)	175 (53.5)	152 (46.5)	-

Data presented as n (%).

* Chi-square test.

The internet was used most via smartphones, with a median time spent online of 6.0 (3.0; 11.0) hours per day. Median smartphone use was 4 (3.0; 8.5) hours among those classified as non-dependent, 6.5 (4.0; 11.2) hours in those with mild dependence, and 10.0 (6.0; 12.7) hours in those with moderate or severe dependence, being significantly higher in the last group (p < 0.001). The median time spent on the internet using a computer was 2.0 (1.0; 4.0) hours. Among those classified as not dependent, the median time spent online on a computer was 2.0 (1.0; 3.0) hours, in those with mild dependence, median time online on a computer was 2.0 (1.0; 5.0) hours and in those with moderate or severe dependence, median time online on a computer was 2.0 (1.0; 4.7) hours. There were no differences in computer usage patterns between non-addicts, mild addicts, moderate addicts, and severe addicts (p = 0.336). We also assessed time spent using tablets, but not enough subjects used this type of device to perform statistical analysis.

The prevalence of IA was 50.8%, considering subjects with mild, moderate, and severe IA. Table 2 lists factors associated with IA. Positive screening for depression and/or anxiety was more frequent among subjects considered as having IA than among those who did not have IA (p = 0.003). There was an association between IA and gaming. Internet addiction was present in 59.1% of the individuals who reported most gaming, while prevalence among those who did not use the internet for gaming was 44.4% (p = 0.010). Among subjects with IA, 70.3% used the internet to access pornographic content, while 47.4% of subjects who accessed this type of content did not have IA. More subjects with IA accessed sexual content than those without IA (p < 0.001). Accessing the internet for work and study content seemed to be a protective factor against IA. Fewer subjects who had IA accessed this type of content (49.8%) than those who without IA (65.2%; p = 0.030).

**Table 2:** Factors associated with Internet addiction in the sample

Variables	Prevalence of Internet addiction (%)	PR (95%CI)	p-value^†^
Gender*			0.633
Male	91 (52.0)	1.05 (0.85-1.31)	
Female	75 (49.3)	1.00	
Age			0.931
14 to 17	74 (51.0)	1.01 (0.81-1.25)	
18 to 20	92 (50.5)	1.00	
Socioeconomic class*			0.689
High (A+B)	46 (53.5)	1.05 (0.74-1.48)	
Middle (C)	95 (49.2)	0.96 (0.70-1.33)	
Low (D+E)	23 (51.1)	1.00	
Screened positive for depression and/or anxiety*			0.003
Yes	146 (55.5)	1.81 (1.23-2.68)	
No	19 (30.6)	1.00	
Content accessed			
Social media*			0.676
Yes	160 (52.3)	1.15 (0.60-2.22)	
No	5 (45.5)	1.00	
Games*			0.010
Yes	97 (59.1)	1.33 (1.07-1.65)	
No	68 (44.4)	1.00	
Information/news*			0.884
Yes	126 (52.3)	1.02 (0.79-1.31)	
No	39 (51.3)	1.00	
Work or study*			0.030
Yes	135 (49.8)	0.76 (0.60-0.97)	
No	30 (65.2)	1.00	
Sexual content*			< 0.001
Yes	45 (70.3)	1.48 (1.21-1.82)	
No	120 (47.4)	1.00	
Total	166 (50.8)	-	-

PR (95%CI) = prevalence ratio (95% confidence interval).

* Missing values.

^†^ Raw Poisson regression scores.

In the adjusted analysis, positive screening for depression/anxiety and content accessed (games, sexual content) remained risk factors for IA, while work/study related access remained as protective factor against IA ( Table 3 ).

**Table 3:** Multivariate analysis (Poisson regression) for factors associated with IA

Variables	PR (95%CI)	p-value
Screened positive for depression and/or anxiety		0.002
Yes	1.86 (1.26-2.75)	
No	1.00	
Content accessed		
Games		0.010
Yes	1.33 (1.07-1.65)	
No	1.00	
Work or study		0.010
Yes	0.74 (0.58-0.93)	
No	1.00	
Sexual content		< 0.001
Yes	1.45 (1.19-1.77)	
No	1.00	

PR (95%CI) = prevalence ratio (95% confidence interval).

## Discussion

This study investigated the prevalence of IA and associated sociodemographic and mental health factors in a school-based sample from Southern Brazil and found a 34.9% prevalence rate of mild IA and a 15.9% prevalence rate of moderate and severe IA, adding up to a total prevalence of 50.8%. There is already a considerable body of studies investigating these prevalence rates in this type of sample, but the majority of these studies were conducted in Asia or Europe.^13 , 24 , 27 , 30 - 34^ As mentioned previously, we only found two studies assessing the same characteristics in Brazil, and neither of them used a probabilistic sample.^18 , 25^ Therefore, the greatest strength of our study is the design that enables us to estimate the IA prevalence rates in this sample in a way that is representative of a whole educational institution.

It is difficult to compare IA prevalence studies due to differences in the instruments used to assess IA, differences in internet access between populations, differences in sample recruitment, differences in categorization of internet usage, and differences in sampling criteria.^12^ Taking these aspects into account, we chose to compare our findings only with other studies written in English, that used Young’s IAT, also known as Young’s Internet Addiction Scale in the literature.^30 , 31^ We only identified one subject classified as a severe internet addict, therefore, for our analysis we have grouped this category together with moderate internet addicts. However, a low prevalence of severe IA is a common finding in the literature. Prevalence rates in the range of 0.7~1.6% have been observed in youth, considering am IAT score of 80 or above.^31 - 33^

Combining moderate and severe IA cases, prevalence rates from 10.5% to 24.8% have been observed, showing that our findings are within the range of what is reported in the literature. Analyzing Asian studies, we found high prevalence rates in a study from South Korea that assessed 1573 adolescents aged 15 and 16 years old.^30^ We also observed that Goel et al.^32^ found the 24.8% prevalence mentioned previously in a sample of 987 teenagers in India aged from 16 to 18 years old. In the same country, an 11.8% prevalence rate was observed in a sample of 552 adolescents aged from 15 to 18 years. In China, assessing larger samples, Tan et al.^35^ found a 17.2% prevalence rate among 1772 adolescents from 13 to 16 years old and Li et al.^33^ studied a sample of 1545 adolescents from 12 to 18 years old, observing a 10.5% prevalence rate. In Turkey, a 16.2% prevalence rate was found in a sample of 468 adolescents aged from 12 to 17 years.^31^ In a setting closer to us, Machado et al.^18^ found a 21% prevalence rate when assessing 91 teenagers from two Brazilians schools.

As stated in the Results section, we only found significant associations when considering all degrees of IA. Thus, analyzing prevalence rates of IA identified by scores over 30 on the IAT, this study found a rate of 50.8% of the sample. Adding together the prevalence rates of studies that reported mild to severe IA rates, we observed rates ranging from 39.5% in South Korea^30^ to 73.3% in Italy.^36^ In Brazil, Méa et al.^28^ also presented data this way, and found a 61.3% prevalence rate in a sample of 150 Brazilian high school students. We can therefore conclude that our prevalence rates are within the range of IA rates reported around the world, even when assessed in different ways.

In our sample, 80.9% screened positive for depression and/or anxiety symptoms, suggesting that further mental health assessments should be carried out in these subjects. Grouping together mild, moderate, and severe IA, the prevalence was higher among those who screened positive for mental health issues (55.5%), but the difference did not attain statistical significance for moderate and severe dependence. A meta-analysis involving eight studies and 1641 patients with IA that investigated the relationship with psychiatric comorbidity found positive associations between addiction and anxiety and/or depression, with prevalence rates for these mental disorders of 20.3% and 14.3% respectively.^37^

It has been established that depressive symptoms are strongly associated with IA in the adolescent population^18 , 30 - 32 , 34 , 35 , 38^ and also with anxiety symptoms.^18 , 31 , 32 , 34 , 39^ It was also observed that both depressive^20 , 33 , 40^ and anxiety^33^ symptoms can possibly predict internet addiction behaviors. According to Li et al.,^33^ who observed that these symptoms were predictive factors, adolescents with internalizing problems (i.e., anxiety, depression) may use the internet as a coping strategy to alleviate their distress. They may seek recreational activities, such as gaming and anonymous communication, among others, to achieve this relief.

In our study, we investigated the main type of internet usage in the sample. We found, in our sample, that playing online games was associated with IA. Other studies have found a similar relationship between online gaming and IA. These researchers also found that another main usage of the internet was for cyber relationships.^23 , 41^ However, that association was not observed in our sample. Internet gaming disorder was included in the current version of the Diagnostic and Statistical Manual of Mental Disorders, 5th edition,^42^ as an important condition, using criteria for substance abuse and gambling disorders. However, it is still regarded as a condition in need of greater study to be considered a formal disorder.^43^ Thus, the relationship between internet use for gaming and IA is not surprising, although we need to be aware that the two conditions are not the same diagnosis, although they are sometimes confused.^44^ Playing online games excessively may be a way to avoid real-life problems through virtual social contact or achievement, since they offer something to people that they cannot find elsewhere.^45^ One hypothesis for the growing use of the internet for online games is that they provide a whole virtual world where socially reclusive adolescents can assume another identity through their online characters, enabling them to be whoever they want to be.

Use of the internet to access pornographic content was significantly higher in subjects who had IA when compared to those who did not (70.3% vs. 47.4%, respectively). This type of access is an important factor related to IA. When an individual compulsively and chronically watches internet pornography, dopamine is continually released into the reward system, stimulating neuroplastic changes that reinforce the experience.^46^ Findings from one study suggest that watching internet pornography exacerbates the risk of IA over time.^47^ Taking this into account, the observation made in the present study is unsurprising and to be expected. There are studies in the literature establishing a clear relationship between internet pornography and IA, but additional studies are still needed to better understand this.^3 , 46 , 47^

Significantly more subjects who did not have IA accessed work and study related content on the internet than those who did. These findings point to a possible factor of protection against IA in subjects who access this type of content. One possible explanation is that people who access these contents do it because they have commitments to their studies or occupation, leaving less time to overuse the internet. There are no other studies in the literature reporting this relationship, therefore, we could not make comparisons. Our findings on the matter suggest a need for more studies to identify the details of this possible protection factor.

Accessing the internet to look for information and using social media were not related to IA in this sample. It might be expected that use of social media would be related to IA, since their use has become a popular tool for social interaction and they constitute an important interactive tool for young people because they provides features that this group seeks, such as connecting people and forming online identities.^48^ From our data, it appears that both groups used the internet to access social media to the same extent, and social media was the type of content most accessed in the sample. One could suggest that social media are so ingrained in the youth lifestyle that they are no longer a factor one can relate to IA, since almost everybody uses them, with stronger relationships restricted to other types of content, that are more specific, less rooted in the core of a group, and accessed by fewer subjects, such as gaming or pornography.

Our sample mainly accessed the internet using their smartphones, with an average of 7.3 hours spent online per day. The rapid increase in smartphone users can be observed all over the world; in some places, users show a clear preference for smartphones over computers.^49 , 50^ Nowadays smartphones are part of people’s routines, and excessive use is a danger, since they are small portable devices that are constantly accessible, making it easier to overuse them.^51^ Many adolescents have their smartphones with them throughout the day, due to their great mobility and functionality.^49^ All internet content and games can be accessed easily through those devices, making this ease of access even more dangerous.^52^ Although use of smartphones provides mobility, overusing them throughout the day can become a problem when users start paying more attention to their devices than to what is happening in their surroundings.^50^

One limitation of our study, which is important to emphasize, is that our findings of depressive and anxious symptoms were obtained using a screening instrument and not with a diagnostic interview or questionnaire. Of course, the prevalence of positive screening was high (80.8%), especially when we know that United Nations estimates mental health disorders among adolescents at around 7~12.7% in Brazil.^53^ We should expect that if another survey based on our paper was conducted with this sample, assessment of mental health problems should be more thorough. It is nevertheless possible to infer that there was an association between IA and altered emotional states with characteristics that are common to depressive or anxious symptoms.

Another limitation of our study, and actually of most of the studies investigating IA, is that the scientific literature on this matter has been hindered by methodological problems. Studies have employed inconsistent and heterogeneous definitions and measurements, because criteria are not established, which affects the results of epidemiological studies.^43^ It is therefore an urgent task for the scientific community to establish consistent and consensual criteria for defining and identifying IA, making it possible to better compare and understand the characteristics and specifics of this addiction in different populations and regions of the world.

## Conclusion

Our study aimed to increase awareness of the presence of IA in this under-investigated population. Further research is needed to confirm the findings of this study and to explore the characteristics of the IA phenomenon in Brazilian adolescents. There are consistent data on IA in other populations, but it is not known if the phenomenon occurs in the same manner and with the same characteristics in this study population. Moreover, the association between this dependency and positive screening for anxiety and/or depression indicates the importance of additional investigation of the problem. Furthermore, new strategies for prevention and treatment of IA, such as psychoeducation and provision of treatment for dependence via the internet, should be developed and adapted to the culture and socioeconomic reality of the population in question. Therefore, through providing these data, we seek to encourage new research, in the hope that it will contribute to improved understanding of this complex phenomenon and the specific nature of the problems associated with internet use among these individuals.
